# Severity Scores in SARS-CoV-2 Infection—A Comprehensive Bibliometric Review and Visualization Analysis

**DOI:** 10.3390/epidemiologia7010008

**Published:** 2026-01-05

**Authors:** Andreea Magdalena Ghibu, Ionela Maniu, Victoria Birlutiu

**Affiliations:** 1 Faculty of Medicine, Lucian Blaga University of Sibiu, 550169 Sibiu, Romania; victoria.birlutiu@ulbsibiu.ro; 2 Infectious Diseases Department, Academic Emergency Hospital Sibiu, 550245 Sibiu, Romania; 3 Faculty of Sciences, Department of Mathematics and Informatics, Research Center in Informatics and Information Technology, Lucian Blaga University of Sibiu, 550024 Sibiu, Romania; 4 Research Team, Pediatric Clinical Hospital Sibiu, 550166 Sibiu, Romania

**Keywords:** COVID-19, severity scores, pandemic, specificity, sensibility

## Abstract

Background/Objectives: Discovered in 2019, COVID-19 spread rapidly worldwide, leading from mild forms of the disease to critical forms or death, predominantly among vulnerable patients. Severity scores help clinicians in stratifying the risk of complications and death among patients diagnosed with SARS-CoV-2 infection. Methods: This study aims to identify the severity scores used in this type of infection, while bibliometric analysis carried out provided a comprehensive overview of global research patterns, trends, and cooperation in scientific literature on the chosen topic. Results: We conducted a literature screening to identify severity scores used in SARS-CoV-2 infection. Scores including CURB-54, COVID-GRAM, NEWS, APACHE II, SOFA, qSOFA, CALL, MuLBSTA, ISARIC 4C, and PADUA were identified with different performance indices. Conclusions: There were different results obtained depending on the geographical area of applicability, patient groups analyzed, and individual patient characteristics.

## 1. Introduction

The new coronavirus, discovered at the end of 2019 in Wuhan, China, caused a large-scale pandemic, spreading quickly around the world. Because of this, the World Health Organization, at the end of January 2020, declared the COVID-19 pandemic a health emergency with international implications. In this context, a key aspect of disease management was the prompt identification of symptoms, early referral to healthcare facilities, and assessment of severity [1]. Severity scores help clinicians by supporting the rapid assessment of patients, evaluating the risk of developing complications, and predicting in-hospital mortality among patients with or without comorbidities and diagnosed with SARS-CoV-2 infection.

The virus’s constant mutations cause a variety of symptoms, with each strain having its own clinical characteristics. Thus, SARS-CoV-2 infection can cause mild forms of the disease to critical forms with severe acute respiratory distress syndrome, thus requiring the use of good predictors for clinical progression from the moment of admission [2]. Furthermore, various risk factors have been identified that are associated with unfavorable outcomes and mortality in patients with SARS-CoV-2 infection. Male gender, age, multiple comorbidities (most commonly cardiovascular disease), and important pulmonary involvement, correlated with significant changes in laboratory parameters, were included [3,4]. Several severity scores have been identified as useful in predicting mortality in COVID-19 infection, including CURB-65, Pneumonia Severity Index, GRAM-COVID, MuLBSTA, APACHE II, SOFA, qSOFA, and PADUA [5,6].

Pneumonia Severity Index (PSI) and CURB-65 are two of the scores recommended for identifying the risk of death and the necessity of hospitalization in patients with pneumonia. The CURB-65 score uses parameters including confusion (1 point), urea > 7 mmol/L > 19 mg/dL (1 point), respiratory rate ≥ 30 breaths/min (1 point), systolic blood pressure < 90 mmHg (1 point), diastolic blood pressure ≤ 60 mmHg, and age ≥ 65 years (1 point). Consequently, a score of 0–1 means that the patient can be treated ambulatory, a score of 2 requires short-term medical monitoring, and a score of 3, 4, or 5 requires hospitalization [7].

Pneumonia Severity Index analyzes patient age, gender, and status as a nursing home resident. Regarding comorbidities, tumor and liver pathology, congestive heart failure, cerebrovascular disease, and renal impairment were included. Clinical parameters are another section of the score that analyzes RR > 30 breaths/min, SBP < 90 mmHg, body temperature < 35 °C or >40 °C, and heart rate > 125 bpm. Biological parameters include arterial pH < 7.35, blood urea > 30 mg/dL, seric sodium < 130 mmol/L, seric glucose > 250 mg/dL, hematocrit < 30%, and partial pressure of oxygen < 60 mmHg or pleural effusion (Appendix A Table A1). Consequently, patients are stratified into five risk groups: group I of outpatients under 50 years old with or without comorbidities and low risk, group II below 70 points with low risk of outpatients, group III between 71 and 90 points with low risk but with possible need of short hospitalization, group IV between 91 and 130 points with moderate risk, and group V above 130 points with high risk, including patients who need hospitalization [3].

MuLBSTA, published in 2019, is a newly developed severity score to stratify the prognosis of viral pneumonia, using six clinical and paraclinical parameters. It quantifies the severity of lung involvement, lymphocyte count ≤ 0.8 × 10^9^/L, presence of bacterial co-infections, smoking status (active or passive), history of hypertension, and age over 60 years (Appendix A Table A2) [8].

Considering the significant coagulation disorders associated with SARS-CoV-2 infection, the PADUA Prediction Score proposes the evaluation of parameters including the presence of active cancer (3 points), history of pulmonary embolism/deep vein thrombosis (3 points), reduced mobility (3 points), diagnosed thrombophilia (3 points), trauma or surgery < 1 month (2 points), age > 70 years (1 point), heart or respiratory failure (1 point), myocardial infarction or ischemic stroke (1 point), acute infection and/or rheumatic disease (1 point), obesity (BMI ≥ 30 kg/m^2^) (1 point), and hormone treatment (1 point). Thus, a PADUA score ≥ 4 points is associated with an increased risk of venous thromboembolism [9].

Another severity score used is GRAM-COVID. This score includes the following parameters: radiological abnormalities, age, presence or absence of hemoptysis, presence of dyspnea, number of comorbidities (between 0 and 5), history of tumors, neutrophil/lymphocyte ratio (N/L), LDH (U/L), and direct bilirubin (mg/dL) [10]. Chronic obstructive pulmonary disease, cardiac diseases, type I and II diabetes, chronic kidney disease, cerebrovascular disease, hepatitis B, and immunodeficiency were included. A low-risk group is identified with a percentage < 1.7%, medium risk between 1.7 and 40.4%, and >40.4%, corresponding to the high-risk group [11].

APACHE II is a score used in Intensive Care Units to assess patients’ health condition using 12 parameters, in addition to an age distribution of patients and an evaluation of their history regarding immunocompromised status or multiple organ failure. This score is performed during the first 24 h after admission in the ICU and is widely used [12].

The SOFA score (Sequential Organ Failure Assessment), first published in 1996, was initially created to assess organ dysfunction in sepsis in the Intensive Care Unit and its applicability was later extended to predict mortality in SARS-CoV-2 infection, evaluating six important systems/organs. Respiratory and cardiovascular system, coagulation, hepato-renal system, and neurological assessment are among these, with 0 to 4 points assigned to each [13]. qSOFA is a shorter version of SOFA score, including a respiratory rate above 22 breaths/min, Glasgow Coma Scale below 15 points, and Systolic Blood Pressure less than or equal to 100 mmHg [14].

In the context of a fast-growing body of research volume on topics related to COVID-19 and the need for rapid and objective tools for assessing the severity of COVID-19 cases, it is difficult to perform a comparative analysis of performance of different severity scores to assess mortality due to COVID-19 across different countries. Bibliometric analysis and visualization networks can bring a new perspective on how the scientific community has approached the topic. Bibliometric analysis can provide a picture of the geographical distribution and intensity of the research within a research field. The present study combines both bibliometric analysis and synthesis of clinical studies to assess their usefulness and accuracy in predicting the prognosis of patients with SARS-CoV-2 infection and the mortality of patients with this type of infection. The network analysis was carried out to provide a comprehensive overview of global research patterns, trends, and cooperation in scientific literature on this topic.

## 2. Materials and Methods

**Figure 1:**
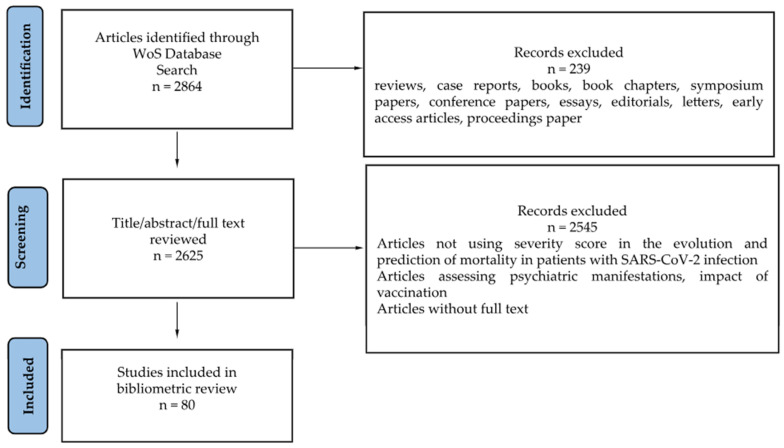
PRISMA flow chart of the study selection process. Out of 2625 reviewed articles, 2330 were excluded based on review of the title and abstract, 295 were analyzed in full text, and 80 articles were included in the bibliometric review.

In June 2025, we conducted a search in the Web of Science Core Collection online database to identify severity scores used in the literature to predict the severity and mortality of patients with SARS-CoV-2 infection. The search formula used in Web of Science was: (COVID 19 OR COVID-19) AND (severity index OR severity score OR “MuLBSTA score” OR “Pneumonia Severity Index” OR “CURB-65” OR “APACHE II” OR “PADUA Prediction Score” OR “Gram-COVID”) AND mortality. The search considered publications that included the above-mentioned terms in their title, abstract, or keywords. The analysis covered the original articles (observational, experimental, and qualitative studies), in English, French, and Spanish, published between 2020 and June 2025. Systematic review articles, case reports, books, book chapters, symposium and conference papers, essays, editorials, and letters were excluded. Authors AMG and IM analyzed the articles (title, abstract, and full text) and discussed any discrepancies that emerged, in order to select the articles relevant for the topic. Only articles including original data for different severity scores and analyzing mortality in the case of SARS-CoV-2-infected patients were considered eligible. Articles containing the established keywords but whose subject matter was focused on (i) comorbidities, laboratory parameters, imaging, and therapy without highlighting the usefulness of a severity score in the evolution and prediction of mortality in patients with SARS-CoV-2 infection, (ii) psychiatric manifestations, (iii) comparative analyses between SARS-CoV-2 infection and other viruses (e.g., influenza) without analyzing mortality or severity scores, or (iv) the impact of vaccination and the applicability of scores in the Emergency Department, were not considered eligible (Figure 1).

VOSviewer software (v.1.6.16, VanEck and Waltman, Center for Science and Technology Studies of Leiden University, Leiden, The Netherlands [15]), a widely used bibliometric visualization analysis tool in medicine and other domains [16,17,18,19,20,21,22,23,24,25], was utilized to visualize collaboration networks and keyword association. Network visualization maps were used for presenting co-authorship and citation maps of countries (using a threshold of (1)) and co-occurrence of terms (using a threshold of (2)). The implicit normalization method (association strength) was considered. The complementary bibliometric indicators h-index (a country’s number of articles (h) that have (at least) h citations) and total link strength (TLS—a measure of a node’s overall connection to all other nodes in the network, calculated by summing up all the link strengths connected to it) were used for country in order to quantify scientific productivity, impact, and collaboration. These indicators are useful in providing deeper insight into the dynamics of scientific collaboration. WordCloud maps were also used to show temporal evolution of terms (extracted after full text analysis of the 80 studies) related to severity scores.

## 3. Results

**Figure 2:**
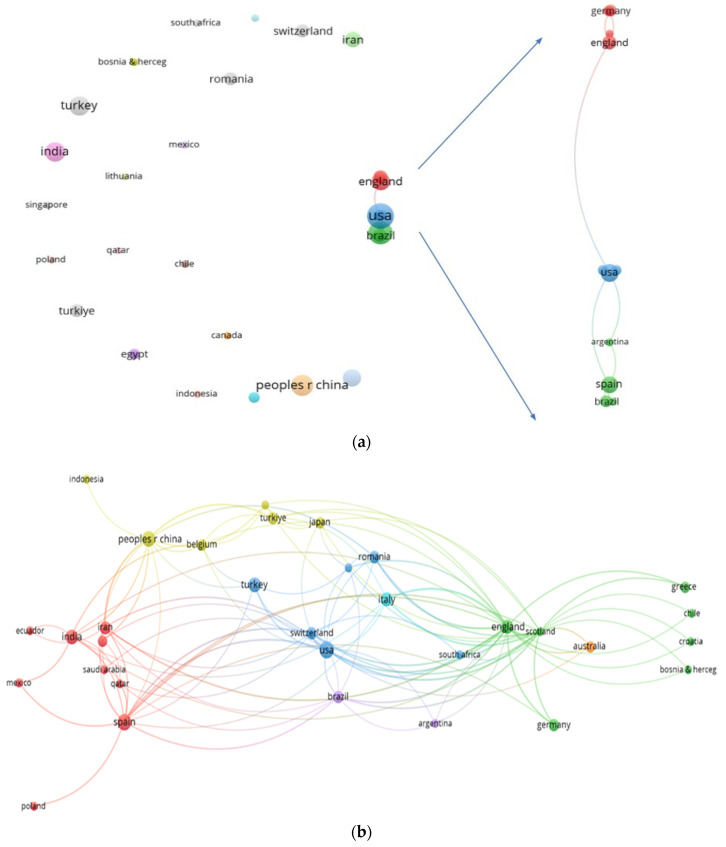
VOSviewer network visualization of (**a**) map of co-authorship between countries; there were 11 interconnected countries: USA, Australia, Japan, England, Scotland, Germany, Greece, Argentina, Spain, Brazil, and Ecuador (right side of the figure), while others are isolated/unconnected countries and did not have international collaboration; and (**b**) map of citations between countries.

There were 80 research articles authored by 779 authors (25 having the maximum number of 2 publications), involving 279 institutions and 34 countries, including the following: USA (10 documents, TLS = 5), Spain (8, TLS = 4), China (7, TLS = 0), India (6, TLS = 0), and Turkey (6, TLS = 0). The biggest h-index was 6 for USA and China, followed by an h-index of 5 for Spain, 3 for India, and an h-index of 2 for Turkey. The hierarchy according to the number of citations is as follows: England (869), Scotland (773), China (442), USA (230), and Spain (145). The network visualization maps between countries’ collaboration and co-citation are presented in Figure 2.

Among the articles with more than 100 citations are the following: (1) Risk stratification of patients admitted to hospital with COVID-19 using the ISARIC WHO Clinical Characterization Protocol: development and validation of the 4C Mortality Score [26], by Knight et al. (with authors from Univ. of Edinburgh, Univ. of Glasgow, Queen Elizabeth Hospital, Univ. of Liverpool, Univ. of Oxford, Imperial College London, Univ. of Birmingham, Univ. of Nottingham, Royal Liverpool Hospital, and Royal Infirmary of Edinburgh), published in BMJ-British Medical Journal (773 citations); (2) Clinical characteristics and outcomes of critically ill patients with novel coronavirus infectious disease (COVID-19) in China: a retrospective multicenter study [27], by Xie et al. (authors from Southeast Univ. Nanjing, Wuhan Jin-Yintan Hospital, Univ. of Science and Technology Wuhan, Wuhan Pulmonary Hospital, Shenzhen Third Hospital, Huangshi Hospital of Chinese Medicine, Capital Medical Univ. Beijing, 900th Hospital of Joint Service Corps of Chinese PLA Fuzhou, Fudan Univ. Shanghai, Guangzhou Medical Univ., Yangzhou Univ., Peking Union Medical College Hospital, Chinese Academy of Medical Sciences Beijing), published in the journal Intensive Care Medicine (129 citations); and (3) Comparing Rapid Scoring Systems in Mortality Prediction of Critically Ill Patients With Novel Corona-virus Disease [28], by Hu et al. (with authors from West China Hospital, China International Emergency Medical Team—Chengdu, Sichuan Univ., Wuhan Univ.), published in the journal Academic Emergency Medicine.

Knight et al. validated the ISARIC 4C score in the UK on a cohort of 22,361 patients, reporting an overall mortality rate of 30.1% between February and June 2020, stratifying patients into four risk groups. An analysis of mortality in these risk groups showed a mortality rate of 31.4% in the high-risk group and 61.5% in the very high-risk group. In addition, performance indicators showed a sensitivity of 99.7% and a specificity of 10.4% of the score in the low-risk group, with a mortality rate of 1.2% in a total of 1650 patients. A comparison was also made between the ISARIC 4C score (AU-ROC: 0.774, 95% CI [0.767, 0.782]) and other predictive models, including qSOFA (AUROC: 0.622 (95% CI [0.615, 0.630]), CURB-65 AUROC: 0.720, 95% CI [0.713, 0.728]), COVID-GRAM (AUROC: 0.706, 95% CI [0.675, 0.736]), A-DROP (AUROC: 0.736, 95% CI [0.728, 0.744]), or NEWS (AUROC: 0.654 95% CI [0.645, 0.662]), underlying the superiority of the score studied [26].

In a study of 733 patients admitted to the Intensive Care Unit, Xie et al. reported a high mortality rate (53.8%) among patients with SARS-CoV-2 infection, where 394 of them died within 28 days of admission. The APACHE II and SOFA scores were applied as soon as the patients were admitted. Moreover, among non-survivors, an APACHE II score of 13 and a SOFA score of 5 were recorded, while among survivors, the scores were 7 and 2, respectively. Regarding adjacent pathology, hypertension and diabetes mellitus were prevalent in both groups. In addition, a large number of critically ill patients developed cardiac injury, evidenced by increases in troponin and acute renal failure. The negative outcome for these patients was explained by persistent lymphopenia, combined with increases in inflammatory markers and hypoxemia [27].

Hu et al., in a retrospective study involving 138 patients, evaluated the predictability of MEWS and REMS scores regarding in-hospital mortality in patients with COVID-19 infection. They were age-stratified into two groups: <65 years and ≥65 years. In the group of patients under 65 years of age, a statistically significant difference was obtained between the MEWS score (AUC: 0.603, 95% CI [0.462, 0.732]) and the REMS score (AUC: 0.863, 95% CI [0.743, 0.941]), *p* = 0.026 < 0.05, compared to the group of patients over 65 years old. Consequently, it emphasized the superiority of the REMS score in predicting mortality in the group under 65 years old and the similar performance of both scores in the group over 65 years old [28]. Furthermore, Table 1 presents details of the most cited studies.

**Figure 3:**
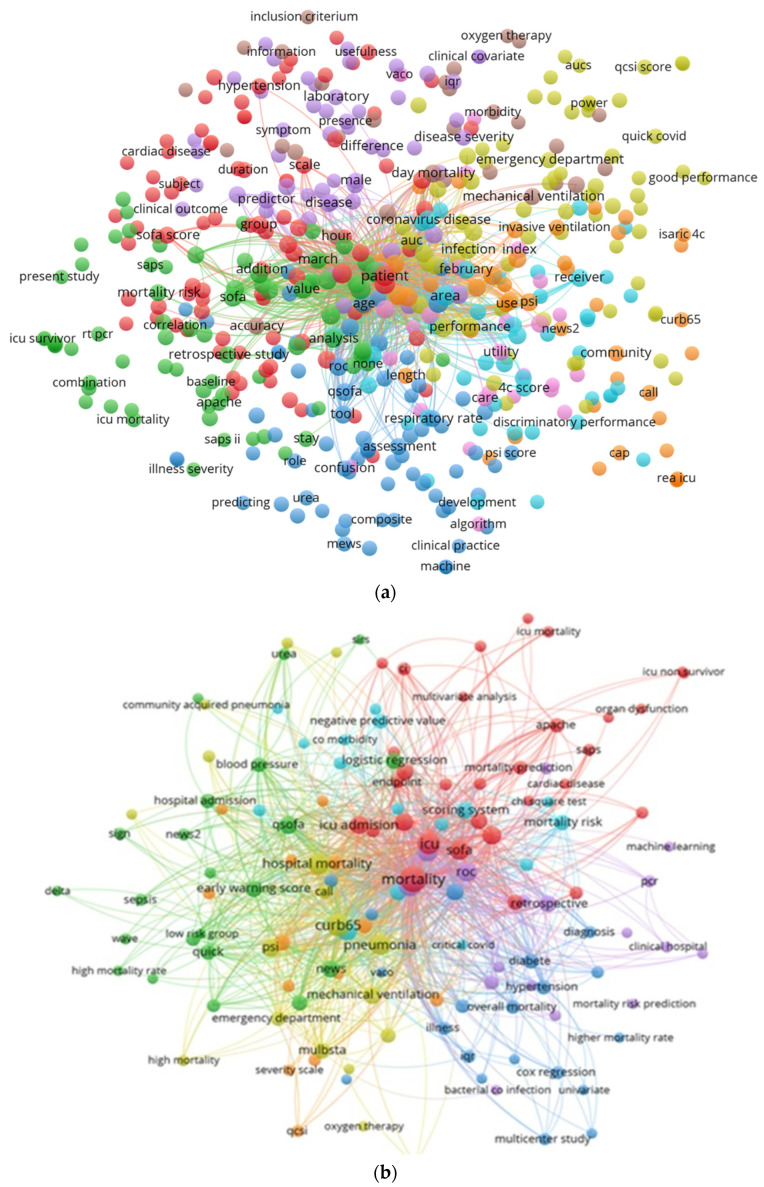
VOSviewer network visualization of terms from titles and abstracts: (**a**) map of terms that appeared at least two times, and (**b**) map of terms based on a thesaurus list of terms.

The co-occurrence map of terms (from title and abstract) is presented in Figure 3. It may be observed that the most frequently encountered scoring-system-related terms were SOFA, CURB-65, QSOFA, NEWS, NEWS2, 4C, QUICK, PSI (Pneumonia Severity Index), MuLBSTA, and APACHE. Among terms related to the outcomes were mortality, overall mortality, hospital mortality, mortality risk, ICU mortality, ICU survivor, morbidity, and hospital admission, while among the data analysis methods, related terms were logistic regression, cox regression, chi square test, univariate and multivariate analysis, ROC, machine learning methods, and accuracy.

**Figure 4:**
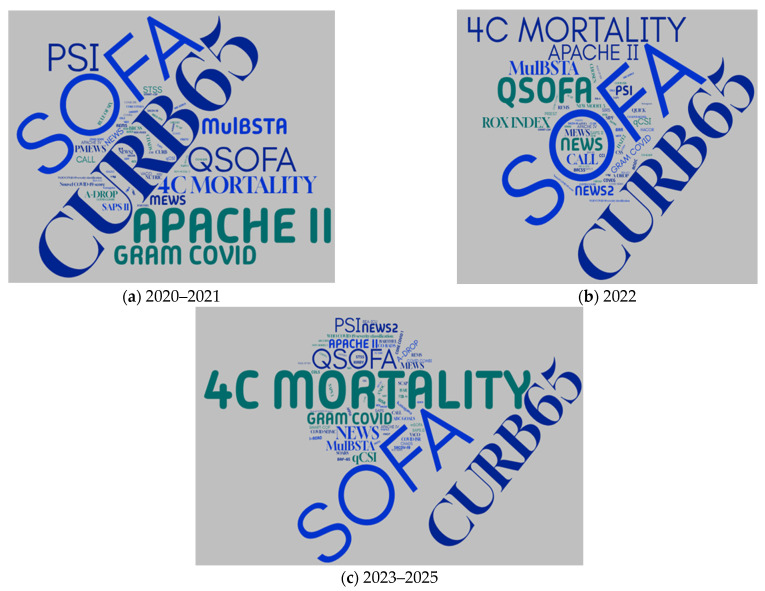
WordCloud graphics showing temporal distribution of scores used for three different time spans: (**a**) 2020–2021 (early COVID-19, validation studies), (**b**) 2022, and (**c**) 2023–2025 (recalibration studies). A threshold of two appearances for a score was considered.

Furthermore, the full text analysis of the 80 retrieved research articles allowed the identification of a larger number of scores used by researchers on this topic. To identify thematic trends and the temporal evolution of interest, we analyzed the temporal distribution of scores used for three different time spans: 2020–2021 (early COVID-19, validation studies), 2022, and 2023–2025 (recalibration studies). The WordCloud from Figure 4a shows the early adopted scores in assessing COVID-19 mortality (SOFA, qSOFA, APACHE, 4C, CURB-65), corresponding to the period 2020–2021. While many early scores remained relevant in 2022 (some scores, like APACHE II, PSI, decrease in their peak usage compared to the 2020–2021 period), it can be noticed that there is an increase in the use of scores like NEWS2, qCSI, and CALL. Also, in the 2022 period, the emergence of new scores can be noticed (ROX INDEX) (Figure 4b). In 2023–2025, there is a drop in the frequency of most scores, perhaps due to the fact that the pandemic evolved and became more manageable (e.g., with better treatments and widespread vaccination) (Figure 4c). The crowded area of scores written in smaller letters in WordCloud figures (scores used with very low frequency: 1–2 instances) indicates a wide variety of approaches and localized preferences in assessing COVID-19 mortality (most did not gain widespread adoption). The scores SOFA, qSOFA, CURB-65, and 4C consistently appear across all three periods, suggesting their enduring relevance, with the last of them (4C) following an upward trend throughout the three periods analyzed and also a good performance in predicting mortality (Table 1).

## 4. Discussion

Given the rapidly expanding volume of COVID-19 research and the urgent need for objective tools to assess case severity, performing a comparative analysis of mortality-predicting severity scores across different countries remains challenging. To address this complexity, the current study employs a dual approach, combining a bibliometric analysis with a synthesis of clinical studies on severity scores used to assess SARS-CoV-2 infection mortality.

The bibliometric analysis showed that USA, Spain, and China were the most productive countries, with USA also leading in terms of collaboration with other countries. This dominance was also encountered in other bibliometric research and could be due to the existence of a large number of researchers and research laboratories, superior (bio)technology, and funds. The lack of collaboration between countries, on the analyzed topic, is emphasized by the network visualization map of co-authorship between countries. The co-occurrence map of terms from titles and abstracts provides a picture of the dominant research themes. The analyzed studies focus mainly on SOFA, CURB-65, qSOFA, NEWS, 4C, PSI, MuLBSTA, and APACHE scores, along with terms related to mortality prediction and analysis methods. Furthermore, the analysis of used scores, extracted after full text analysis, offered deeper insights on thematic trends and the temporal evolution of interest. The scores SOFA, qSOFA, CURB-65, and 4C consistently appeared across all three periods of analysis. Notably, the 4C score followed an increasing trajectory across these periods. An explanation could be that it uses few parameters that require invasive identification techniques, giving it methodological advantages independently of the pandemic phase.

Several severity scores have been identified for assessing the risk of death in patients diagnosed with SARS-CoV2 infection. A study conducted on a cohort of 247 patients in Ecuador, Carriel et al., emphasizes that although CURB-65 was designed for bacterial pneumonia, it can be useful in predicting 30-day mortality in COVID-19 infection. A score above two points had a sensitivity of 84% and a specificity of 54% (AUC: 0.72, 95% CI: [68, 86], *p* < 0.001) [39]. In a French comparative study, on a quasi-similar group of patients, the authors concluded that although it is a good predictor of mortality, it cannot objectively establish the necessity of hospitalizing these patients or treating them ambulatory. This aspect is based on epidemiological and severity differences between bacterial and viral pneumonia [39,40].

The Pneumonia Severity Index provides complexity in the parameters evaluated. Bradley et al., analyzing a cohort of 8081 patients, identified a slight superiority in predicting the mortality of PSI compared to CURB-65 either in the cohort of patients with SARS-CoV-2 infection (AUC: 0.82 vs. 0.879) and in those with other infections (AUC: 0.79 vs. 0.75) with similar specificity and sensitivity [37,38]. This fact could be explained by the assessment of comorbidities and age, two important risk factors in the mortality of patients with COVID-19 infection [38]. These results are similar to those from a multicenter, retrospective study in Spain, in a cohort of 10,238 patients (AUC: 0.835 vs. 0.825) [2]. Procoagulant status in SARS-CoV-2 infection, with microthrombosis formation, was often described in non-survivor patients. Moreover, it was attempted to improve the predictability of these two scores by combining d-dimers and procalcitonin, but the results obtained were not significantly improved [38]. Lactate association with the parameters analyzed in the CURB-65 score did not improve its predictive value [41].

The MuLBSTA score was designed to estimate the risk of mortality at 90 days [8]; consequently a score ≥ 12 points is associated with a higher risk of mortality. Preetam et al. applied this score to a group of 122 patients, obtaining a similar prediction of mortality at 90 days and 14 days, at a score ≥ 12 points, but with an important limitation related to the small number of patients enrolled [42]. In a study conducted in a group of 208 patients, Ronda et al. performed a comparative analysis between the Pneumonia Severity Index (AUC: 0.824), CURB-65 (AUC: 0.821), MulBSTA (AUC: 0.715), and GRAM-COVID (AUC: 0.857) in mortality among patients with SARS-CoV-2 infection, showing that the GRAM-COVID score obtained the best ROC curve, sensitivity, specificity, and predictive values.

The CALL score is another tool identified to stratify the mortality risk of patients with SARS-CoV-2 infection. It uses parameters such as age over 60 years old, absolute lymphocyte count below <1000/L, LDH value, and the presence of comorbidities. The pathologies evaluated included cardiovascular diseases, pulmonary pathology, metabolic diseases, HIV infection, hepatic pathology, and tumors diagnosed in the last 6 months, dividing patients into three groups: class A between 4 and 6 points, class B between 7 and 9 points, and class C with 10 points) [31,32]. Ucan et al., in a comparative study in a cohort of 296 patients, showed that the A-DROP score (AUC: 0.875) and PSI (AUC: 0.873) were better at predicting mortality in patients with SARS-CoV-2 infection, as well as CALL, CURB-65, and GRAM-COVID scores, which had a quasi-similar 95% CI. It also emphasizes that no mortality was identified in the low-risk group of the CALL score. Regarding infection severity prediction, all scores had lower predictive values. The CURB-65 score performed best in this category with an AUC of 0.737, followed by the CALL score with an AUC of 0.693. The CALL score was also reported to be a better predictor in models adjusted for age and comorbidities, as well as in unadjusted models [43,44]. In terms of the ability to identify patients at risk of developing a severe form of the disease in the early stages, the performance of the CALL score was satisfactory, which could be explained by the time between the patient’s admission and the need for invasive ventilation [45].

The A-DROP score is a modified version of the CURB-65 score, using the following parameters: age with gender differences (above 70 years in men or 75 years in women), Blood Urea ≥ 21 mg/dL or dehydration, SaO_2_ ≤ 90 mmHg/PaO_2_ ≤ 60 mmHg, systolic blood pressure ≤ 90 mmHg, and consciousness. Its predictive value is reported to be similar to that of the CURB-65 score and the Pneumonia Severity Index [46].

APACHE II provides information related to the assessment of disease severity and the risk of death in patients [12]. Wang et al., in a study conducted in a group of 235 patients, highlights the fact that the accuracy of the MEWS score (AUC: 0.913) is comparable to APACHE II (AUC: 0.937), PSI (AUC: 0.927) or SOFA (AUC: 0.926) in terms of mortality among the elderly. The MEWS score can be performed during the first few minutes of admission using five parameters, including systolic blood pressure, heart rate, temperature, respiratory rate, and level of consciousness, making it easy to apply compared to other scores [33]. Covino et al. emphasize in a study conducted among patients over 80 years old with COVID-19 infection, the accuracy of the APACHE II score compared to the SOFA or CURB-65 scores, concluding that, given the severity of their symptoms and the severity of lung damage, being elderly, having dementia, and having limited daily activity are important negative prognostic factors [35]. Comorbidities in elderly patients and others were also an important factor in patient outcomes. The reviewed articles mentioned cardiovascular disease, diabetes, pulmonary and neurological disease, being overweight, kidney disease, and neoplasia as the most common conditions found in the cohorts studied [9,13,35,47]. ISARIC 4C is a score that considers parameters that are easy to use at the time of admission and does not depend on imaging assessment. This score stratifies patients into four risk groups for in-hospital mortality in patients with SARS-CoV-2 infection, and was used for the first time in the United Kingdom. It uses parameters like age, gender, number of comorbidities, respiratory rate, oxygen saturation, Glasgow coma score, Blood Urea, and CRP (Appendix A Table A3). Chronic cardiovascular and pulmonary disease, severe chronic kidney disease, moderate to severe liver disease, dementia, insulin-requiring type II diabetes mellitus or diabetes treated with oral antidiabetic drugs, tumors, HIV, and obesity were included. A score between 0 and 3 points classifies patients as having a minor risk of mortality, between 4 and 8 points as having an intermediate risk, a high risk at a score of 9–14 points, and a very high risk at a score of over 15 [47,48].

Albai et al. showed, in a study, the efficiency of this score in predicting mortality in patients with diabetes mellitus and hospitalized with SARS-CoV-2 infection, especially in patients with uncontrolled blood sugar levels or complicated diseases. Furthermore, at an ISARIC-4C score above 14 points, an increased mortality rate was reported with an overall mortality in the study group of 25.15% [47]. Durie et al. compared the ISARIC-4C score with the APACHE II score and concluded that the ISARIC-4C score overestimated mortality in the Australian population studied, who were hospitalized in the Intensive Care Unit. In addition, its performance was weaker compared to the APACHE II score (AUC: 0.791 vs. AUC: 0.810), citing these differences as being based on possible host factors, disease, or systemic factors [49], compared to the original study in the United Kingdom.

Gruyters et al. reported that an increased SOFA score upon admission of patients with COVID-19 to the ICU did not show statistical differences between the two groups, i.e., between non-survivors and survivors, in contrast to other studies that analyzed similar aspects [14,50,51]. This score was reported to have good specificity (over 80%) in predicting mortality in patients with COVID-19 and cardiovascular comorbidities, diabetes mellitus, chronic kidney disease, or patients with tumor pathology [32,33,52].

Such a study is the one led by Citu et al., who validated this score in a cohort of 133 patients, reporting it as an extremely useful tool in predicting patients requiring admission to intensive care (AUC: 0.800). A comparative analysis was also performed with the qSOFA score, concluding that the SOFA score has higher sensitivity (94.4% vs. 61.1%) but lower specificity, with both scores predicting mortality at a cut-off value of 2 (Youden Index) [53]. Furthermore, compared to other scores (4C, NEWS, or CURB-65), the accuracy of the SOFA score regarding mortality in patients with COVID-19 was lower (AUC: 0.800 vs. 0.818 vs. 0.861 vs. 0.801) [54]. Such a predictive value between the SOFA score, 4C score, APACHE II, and SAPS was also reported in the study conducted by Vicka et al. [55]. Regarding the accuracy of the qSOFA score, although a good predictor of mortality, it showed lower accuracy compared to other scores. Alencar et al. performed such a comparison with SIRS [56].

Another score used in sepsis, whose predictive value has also been studied in COVID-19 infection, is the NEWS 2 score. It uses six clinical parameters, including respiratory rate, oxygen saturation, systolic blood pressure, heart rate, consciousness, and temperature, with two additional points added if the patient requires oxygen therapy. Moreover, a score of 5–6 points could indicate a possible deterioration in the patient’s health, requiring a clinician’s intervention [4]. Prim et al. recognized as good predictors in the studied group both the NEWS II score (AUC: 0.774; 95% CI [0.73, 0.82]), *p* < 0.001), and the ISARIC-4C score (AUC: 0.771; 95% CI [0.73, 0.81], *p* < 0.001). The mortality rate was 8%, with better sensitivity of the NEWS II score at the five-point threshold for urgent response [57]. Comparing the specificity and sensitivity in predicting in-hospital mortality of the NEWS score with complex scores including APACHE II, SOFA, and qSOFA, their high specificity is evident (above 70%), especially among men with chronic heart disease, diabetes, or neoplasms [52,58,59,60].

Other predictability scores have also been identified, including SCAP and SMART-COP. These scores were designed to decide when admission to Intensive Care Unit is needed, when mechanical ventilation should start, and when vasopressor medication should be given. According to a study conducted by Peñafiel et al., the two scores showed ROC curves similar to those of CURB-65, CALL, or GRAM-COVID in terms of the risk of complications and death, but further studies are needed in this regard [61].

The study has several limitations. We included only articles from the Web of Science database. A more detailed analysis could include searching multiple databases such as Scopus, PubMed, Google Scholar, etc. The study used VOSviewer software to perform bibliometric analysis; additional bibliometric tools (Bibliometrix, CiteSpace, etc.) could be used to offer expanded analysis. Also, future research directions should take into consideration the development of hybrid AI-based scoring systems, combining clinical and genetic biomarkers with multicentric validation across populations.

## 5. Conclusions

The study presents a combination of both bibliometric analysis and a synthesis of clinical studies on severity scores used to assess mortality in SARS-CoV-2 infection. There is an imperative need for improvement in terms of international collaboration. The network visualization map of terms and full text analysis offered a picture of dominant research themes and deeper insights on thematic trends and the temporal evolution of interest.

Severity scores are effective tools for predicting mortality in COVID-19 infection, with their prediction accuracy being influenced by numerous factors, including demographic data, comorbidities, or laboratory parameters. The differences between results stem from limitations related to either the number of patients included in the study or their use in isolated centers, which did not allow data homogenization and the highlighting of a single score. The simplicity and the time needed to perform these scores are important aspects that influence the decision to choose them. Also, association parameters including oxygen saturation, PaO_2_/FiO_2_, respiratory index, interleukin 6 value, or age above 60 could improve the prediction of these scores in order to assess the infection severity.

## Figures and Tables

**Table 1:** Top most cited studies.

Article Title Reference No. of Citations	First Author Year of Publication Country	Number of Patients Enrolled	Severity Scores	AUC Specificity Sensitivity
Risk stratification of patients admitted to hospital with COVID-19 using the ISARIC WHO Clinical Characterization Protocol: development and validation of the 4C Mortality Score [26] 773	Stephen R Knight 2020 England	35,463	4C A-DROP GRAM COVID NEWS qSOFA SOFA	AUC: 0.774 AUC: 0.736 AUC: 0.706 AUC: 0.654 AUC: 0.622 AUC: 0.614
Clinical characteristics and outcomes of critically ill patients with novel coronavirus infectious disease (COVID-19) in China: a retrospective multicenter study [27] 129	Jianfeng Xie 2020 China	733	APACHE II SOFA	
Comparing Rapid Scoring Systems in Mortality Prediction of Critically Ill Patients With Novel Coronavirus Disease [28] 110	Hai Hu 2020 China	105	MEWS REMS	AUC: 0.677 AUC: 0.841
Predictive performance of SOFA and qSOFA for in-hospital mortality in severe novel coronavirus disease [14] 95	Sijia Liu 2020 China	127	SOFA qSOFA	AUC: 0.890 AUC: 0.742
A novel severity score to predict inpatient mortality in COVID-19 patients [29] 91	David J. Altschul 2020 USA	2356	Nouvel COVID-19 score	AUC: 0.798
Utility of established prognostic scores in COVID-19 hospital admissions: multicentre prospective evaluation of CURB-65, NEWS2 and qSOFA [30] 67	Patrick Bradley 2020 England	830	CURB-65 (<2 points) NEWS II (<5 points) qSOFA (<2 points)	AUC: 0.76/0.86/0.48 AUC: 0.78/0.92/0.32 AUC: 0.66/0.92/0.31
Mortality Predictive Value of APACHE II and SOFA Scores inCOVID-19 Patients in the Intensive Care Unit [31] 65	Mohammad Taghi Beigmohammadi 2022 Iran	259	APACHE II SOFA	ROC: 0.73 ROC: 0.8947
The prognostic value of the SOFA score in patients with COVID-19: A retrospective, observational study [32] 48	Zheng Yang 2021 China	117	SOFA	AUC: 0.995/95.40/100
Severity Scores in COVID-19 Pneumonia: a Multicenter, Retrospective, Cohort Study [2] 46	Arturo Artero 2021 Spain	10,238	PSI CURB-65 MulBSTA qSOFA	AUC: 0.835 AUC: 0.825 AUC: 0.715 AUC: 0.728
The utility of MEWS for predicting the mortality in the elderly adults with COVID-19: a retrospective cohort study with comparison to other predictive clinical scores [33] 41	Lichun Wang 2020/ China	235	APACHE II SOFA MEWS PSI CURB-65 qSOFA	AUC: 0.937/87.4/91.9 AUC: 0.926/89.4/81.1 AUC: 0.913/94.5/67.6 AUC: 0.927/86.4/91.9 AUC: 0.845/73.7/83.8 AUC: 0.886/95.0/73.0
Mortality prediction model for the triage of COVID-19, pneumonia, and mechanically ventilated ICU patients: A retrospective study [34] 41	Logan Ryan 2020 USA	114	qSOFA CURB-65 MEWS	AUC: 0.722 AUC: 0.751 AUC: 0.797
Predictors of in-hospital mortality AND death RISK STRATIFICATION among COVID-19 PATIENTS aged ≥ 80 YEARs OLD [35] 40	Marcello Covino 2021 Italy	239	NEWS CURB-65 APACHE II	AUC: 0.649/52/69 AUC: 0.591/78/32 AUC: 0.651/67/62
Performance of the quick COVID-19 severity index and the Brescia-COVID respiratory severity scale in hospitalized patients with COVID-19 in a community hospital setting [36] 37	Guillermo Rodriguez-Nava 2021 USA	313	CURB-65 qCSI BRCSS	AUC: 0.781 AUC: 0.711 AUC: 0.633
Community-acquired pneumonia severity assessment tools in patients hospitalized with COVID-19: a validation and clinical applicability study [37] 36	Felippe Lazar 2021 Brazil	1363	CURB CURB-65 qSOFA PSI GRAM COVID CALL 4C	AUC: 0.71/0.26/0.96 AUC: 0.74/0.53/0.84 AUC: 0.63/0.86/0.34 AUC: 0.79/0.49/0.9 AUC:0.77/0.37/0.91 AUC: 0.71/0.09/0.99 AUC: 0.78/0.09/0.99
Pneumonia Severity Index and CURB-65 Score Are Good Predictors of Mortality in Hospitalized Patients With SARS-CoV-2 Community-Acquired Pneumonia [38] 33	James Bradley 2022 USA	8081	PSI CURB-65	AUC: 0.82/0.66/0.83 AUC: 0.79/0.61/0.83

## Data Availability

Data are contained within the article.

## Abbreviations

The following abbreviations are used in this manuscript:

SOFA	Sequential Organ Failure Assessment
qSOFA	Quick Sequential Organ Failure Assessment
MEWS	Modified Early Warning Score
REMS	Rapid Emergency Medicine Score
APACHE II	Admission Acute Physiologic Assessment and Chronic Health Evaluation II
NEWS	National Early Warning Score
SAPS	Simplified Acute Physiology Score
ICU	Intensive Care Unit
HIV	Human Immunodeficiency Virus
RR	Respiratory Rate
SBP	Systolic Blood Pressure
LDH	Lactate dehydrogenase
CRP	C-Reactive Protein

## Appendix A

**Table A1:** Pneumonia Severity Index Score.

		Points
Characteristics	Age	
	Male	Years
	Female	Years − 10
	Nursing home resident	Years + 10
Comorbidities	Tumor	30
	Liver disease	20
	Congestive heart failure	10
	Cerebrovascular disease	10
	Kidney disease	10
Physical examination	Altered mental status	20
	Respiratory rate ≥ 30 breaths/min	20
	Systolic blood pressure < 90 mmHg	20
	Temperature < 35 °C or >40 °C	15
	Heart rate> 125 bpm	10
Laboratory tests	Arterial pH < 7.35	30
	Blood Urea > 30 mg/dL	20
	Sodium < 130 mmol/L	20
	Glucose > 250 mg/dL	10
	Hematocrit < 30%	10
	Partial pressure of oxygen < 60 mmHg	10
	Pleural effusion	10

**Table A2:** MuLBSTA score.

Characteristics	Yes	No
Multilobar involvement	+5	0
Lymphocyte count ≤ 0.8 × 10^9^/L	+4	0
Bacterial infection	+4	0
Smoking status		
Active smoker	+3	0
Prior smoker	+2	0
History of hypertension	+2	0
Age ≥ 60 years	+2	0

**Table A3:** ISARIC-4C Score.

Characteristics		Points
Age	<50 years	0
	50–59 years	2
	60–69 years	4
	70–79 years	6
	>80 years	7
Gender	Male	1
	Female	2
Comorbidities	0	0
	1	1
	≥2	2
Respiratory rate (breaths/min)	<20 breaths/min	0
	20–29 breaths/min	1
	≥30 breaths/min	2
Oxygen saturation	>92%	0
	<92%	2
Glasgow score	15	0
	<15	2
Blood Urea (mmol/L)	<7	0
	7–14	1
	>14	3
CRP (mg/L)	<50	0
	50–99	1
	≥100	3
